# Supplementary material for: The Relationship between COVID-19 Protection Behaviors and Pandemic-Related Knowledge, Perceptions, Worry Content, and Public Trust in a Turkish Sample
Source: Vaccines (Basel). 2022 Nov 27;10(12):2027. doi: 10.3390/vaccines10122027 (PMC9784616; doi:10.3390/vaccines10122027)
Supplement: Supplementary file 1 [file vaccines-10-02027-s001.zip › vaccines-2013020-SM.pdf]

Supplemantary Electronic Material 1

The questions used to probe pandemic-related emotions and worries

Emotions	Scales
Explanation:<br>Please choose one option per row below.<br>The novel coronavirus to me feels …….	
Close to me ----------------------Far away from me	1- Very close------------------------ 7- Too far away
Spreading slowly ------------------Spreading fast	1-Very slowly-------------------------7- Very fast
Fear-inducing -------------- -----Not fear-inducing	1-Not fear-inducing at all-----7- Extremely fear-inducing
Media hyped ---------------------Not media hyped	1-Media hyped at all-------------7-Not media hyped
Worrying -----------------------------Not worrying	1-Not worrying at all-----------------7-Worrying a lot
Something that makes me feel helpless - Something I am able to combat	1- Something I am able to combat ---7-Extremely helpless
Stressful ---------------------------------Not stressful	1-Not stresfull at all------------7- Extremely stresfull
Worry Domain Questionnaire	Scales
Explanation:<br>Crises often involve fears and worries. Please let us know: At the moment, how much do you worry about:	
Losing someone I love	1- Don’t worry at all --- ----------------7- worry a lot
Health system being overloaded	1- Don’t worry at all --- ----------------7- worry a lot
My own mental health	1- Don’t worry at all --- ----------------7- worry a lot
My own physical health	1- Don’t worry at all --- ----------------7- worry a lot
My loved ones’ health	1- Don’t worry at all --- ----------------7- worry a lot
Restricted liberty of movement	1- Don’t worry at all --- ----------------7- worry a lot
Loosing vacation opportunities	1- Don’t worry at all --- ----------------7- worry a lot
Small companies running out of business	1- Don’t worry at all --- ----------------7- worry a lot
Economic recession in my country	1- Don’t worry at all --- ----------------7- worry a lot
Restricted access to food supplies	1- Don’t worry at all --- ----------------7- worry a lot
Becoming unemployed	1- Don’t worry at all --- ----------------7- worry a lot
Not being able to pay my bills	1- Don’t worry at all --- ----------------7- worry a lot
Not be able to visit people who depend on me	1- Don’t worry at all --- ----------------7- worry a lot
